# National centralized drug procurement and health care expenditure of households—micro-evidence from CFPS

**DOI:** 10.3389/fpubh.2024.1405197

**Published:** 2024-08-19

**Authors:** Xin Li, Ran Tao, Yuning Jin, Na Li

**Affiliations:** ^1^School of Finance, Capital University of Economics and Business, Beijing, China; ^2^Banking Department Under the Headquarters, China Power Finance CO. LTD., Beijing, China; ^3^School of Business, The University of Sydney, Sydney, NSW, Australia

**Keywords:** national centralized drug procurement (NCDP), household health care expenditure, health care policy reform, household economic, CFPS

## Abstract

**Objective:**

This paper utilizes data from the China Family Panel Studies (CFPS) to evaluate the impact of the “4 + 7” National Centralized Drug Procurement (NCDP) on *Per Capita* Household Health Care Expenditure (PCHHCE).

**Methods:**

The study applies the Differences-in-Differences (DID) methodology to analyze the effects of NCDP. Various robustness tests were conducted, including the Permutation test, Propensity Score Matching, alterations in regression methodologies, and consideration of individual fixed effects.

**Results:**

Research indicates that the implementation of NCDP led to a reduction of 10.6% in PCHHCE. The results remained consistent across all robustness tests. Additionally, the research identifies diversity in NCDP effects among various household characteristics, with a more significant impact on households residing in rural regions of China, enrolled in Basic Medical Insurance for urban and rural residents and urban workers, and having an income bracket of 25–75%.

**Conclusion:**

These findings carry policy implications for the future expansion and advancement of NCDP in China. The study highlights the effectiveness of NCDP in reducing healthcare expenditures and suggests potential areas for policy improvement and further research.

## 1 Introduction

Over time, the persistent problem of excessively high pharmaceutical prices has been a central issue in China’s healthcare system reform. Despite the ongoing implementation of policies aimed at tackling this issue, their impact has been limited. As a result, the general public continues to grapple with expensive medical consultations and high medication costs that remain unresolved. Since 2015, China has implemented drug procurement policies, including the “Opinions on Further Standardizing the Centralized Procurement of Medicines in Medical Institutions” and the “Guidance on Enhancing the Centralized Procurement of Medicines in Public Hospitals,” aiming to overhaul the medicine procurement process and bidding methods. In 2018, the government pinpointed four municipalities directly under its jurisdiction—Beijing, Shanghai, Chongqing, and Tianjin—and seven other cities—Shenyang, Dalian, Xiamen, Guangzhou, Shenzhen, Chengdu, and Xi’an—as the pilot cities for the “4 + 7” National Centralized Drug Procurement (hereafter referred to as NCDP). Enterprises participating in the NCDP and meeting the procurement requirements are expected to propose 60 to 70% of the aggregate annual drug consumption of all public medical institutions within the pilot cities as the estimated total procurement volume stipulated in the “4 + 7” Cities Centralized Drug Procurement Document. In December 2018, the announcement of drug tender prices under the “4 + 7” National Centralized Drug Procurement (NCDP) policy had a substantial impact on pharmaceutical prices. The price reductions exceeded expectations, with winning drugs’ average price dropping by 52% and the maximum reduction reaching 96%. For instance, the price of fosinopril sodium tablets, manufactured by Bristol-Myers Squibb and holding an 82.81% market share, decreased by 69.90% after inclusion in the NCDP (1, 2).

The primary objective of China’s government healthcare cost reform is to alleviate the actual medical expenditure burden on residents. Consequently, various related policies are often integrated to optimize efficiency. In this implementation process, the National Centralized Drug Procurement (NCDP) policy plays a pivotal role in reducing residents’ medical expenses, particularly when combined with medical insurance (3). However, within the current medical insurance framework, the disparity exists in residents’ actual medical costs, with lower-income groups facing lower reimbursement ratios compared to higher-income groups. This inequity in medical resource allocation is especially noticeable among minors and middle-aged to older adult populations in rural areas. The NCDP, functioning as an ex-ante compensation mechanism to alleviate residents’ medical expenses related to pharmaceutical costs, partly mitigates the inequality arising from the ex-post compensation mechanism within China’s current medical insurance system (4–11).

Further research is required to prove that the objectives of the NCDP have been achieved during its year-long pilot phase, requiring both quantitative and qualitative investigation. This paper aims to analyze, using theoretical and empirical approaches, the impact and efficacy of the NCDP on *Per Capita* Household Health Care Expenditure (PCHHCE). This study is of significant importance for the government in further optimizing healthcare cost reforms, improving residents’ welfare, and promoting healthcare equity among different groups.

In comparison to earlier literature, this paper’s contribution mainly includes two aspects. Firstly, leveraging microdata, we evaluate the impact of the NCDP on PCHHCE in pilot cities. This analysis utilizes a panel derived from the CFPS questionnaire spanning three periods from 2016 to 2020, allowing for a more refined understanding of the NCDP’s effect on PCHHCE. This study bridges the research gap regarding actual household expenditure under NCDP from an empirical perspective. Secondly, it conducts an analysis of the NCDP’s impact on households with diverse characteristics through a quantitative assessment of heterogeneity, thereby furnishing a more comprehensive evaluation of the policy’s fairness.

The rest of this paper is structured as follows: Section 2 offers an extensive literature review, while Section 3 delineates the data sources and processing methods employed in this study. Section 4 presents the quantitative analysis results and subsequent discussion. Lastly, Section 5 concludes with the study’s findings and policy recommendations.

## 2 Literature review

According to Wang and Yang's (12) general equilibrium theoretical model of healthcare expenditure-health investment, residents’ medical demand is influenced by their health status, while medical expenditure depends on the quantity of medical demand and the prices of medical products and services. As residents age and self-assessed health deteriorates, the quantity of medical demand increases, leading to a rapid rise in total medical expenses. The relative prices of medical products and services in the current period affect the trade-off between residents’ current health investments and future medical expenditures. The healthcare insurance system, as a crucial component of China’s public health and social security, is the primary means to address residents’ medical costs. It partially smooths the gap between current health investments and future medical expenses, reduces the relative prices of healthcare services, lowers expectations of future medical expenditures, and thereby stimulates current healthcare demand. However, current research suggests that healthcare insurance policies relying on ex-post compensation may lead to inequality of opportunity (13, 14).

To address the above issues, the government introduced the NCDP, aiming to reduce overall healthcare costs for the public. NCDP primarily operates through ex-ante compensation, which involves indiscriminately lowering the cost of medical drugs. According to the price elasticity of demand theory by Bhattacharya et al. (15), the price reduction caused by generic drugs will transfer the surplus from producers to consumers. NCDP can reduce healthcare costs at the source. However, different opinions have emerged in the academic community regarding the implementation of NCDP.

Regarding the impact of the NCDP on residents’ medical expenditures, Tan et al. (14) argue that the synergy between medical insurance and the NCDP can adjust the drug supply guarantee system, thereby enhancing the cost-reduction effect of medical insurance and reducing household expenditures. Tang et al. (3), through a study based on data from local hospitals, found that the NCDP led to a decrease in *per capita* total medical expenses and *per capita* drug expenses for hospitalized patients by 644.58 yuan and 300.19 yuan, respectively. However, the NCDP prioritized reducing total medical expenses for middle-aged and older adult residents in urban areas but did not improve internal group inequalities or health disparities (7). In an analysis of local hospital data, Zhi et al. (11) found that the NCDP for psychiatric specialty hospitals from 2019 to 2022 effectively reduced the drug cost burden for mental illness patients and improved the efficiency of medical insurance funds. Xu et al. (10) discovered through a survey of inpatient cardiology patients that the NCDP reduced individual payment costs for patients and increased the payment rate of medical insurance funds. The limited daily cost (DDDc) of EGFR-TKI drugs significantly decreased, usage intensity increased (DDDs), procurement amounts significantly rose, and patient economic burdens were alleviated (16). The DDDs of oral hypoglycemic drugs increased to varying degrees, while drug unit prices and usage amounts proportionally decreased (17). In a survey conducted by Tao et al. (8), 80.9% of patients believed that NCDP drugs were much cheaper than their previous medications. Wen et al. (9), using interrupted time series (ITS) analysis, found that under the “4 + 7” NCDP, the daily average cost of SSRIs significantly decreased by 2.93 yuan, improving the affordability of drugs.

In terms of the impact of the NCDP on healthcare institutions, Dong et al. (18) argue that its implementation will compel public hospitals to reform, reduce excessive medical practices, and promote more rational drug use. However, it may also lead to decreased income for hospitals and healthcare personnel. Zhu et al. (2) found that the NCDP effectively lowers drug prices, resulting in a reduced proportion of drug-related income in healthcare service revenues and affecting the income structure of healthcare institutions. Nevertheless, the NCDP faces challenges, such as limited category choices, which hinder patients from seeking diversified treatment options. Analyzing data from seven low-income countries with different drug procurement systems, including the Philippines, Senegal, and Serbia, (19) discovered that centralized drug procurement allows the public sector to obtain a 15% price discount.

Regarding the impact of the NCDP on pharmaceutical companies’ production and pricing, Dubois et al. (20) suggest that its influence on product prices depends on market concentration and competition levels. Higher market concentration leads to a smaller impact from the policy. Toulemon (21) found that France’s NCDP for innovative high-priced drugs has a greater effect on oligopolistic drug prices than on monopolistic prices. Wang et al. (22) observed a decreasing trend in the price index of substitute drugs for tendered drugs over time. Zhu et al. (23) suggested that the NCDP, through limits on procurement shares, reduces the likelihood of collusion among companies and lowers prices within collusions. The higher the market concentration and the frequency of drug tendering, the smaller the policy’s price reduction effect. Wang et al. (22), based on pharmaceutical sales data and annual reports of winning companies, found that most drugs experienced a significant decrease in sales after being included in the NCDP, especially for original manufacturers. Winning companies have monopolized the market for generic drugs.

Some researchers argue that the NCDP may have certain negative impacts. For example, the implementation process lacks supervision, causing healthcare institutions to underreport drug demand to avoid economic losses, resulting in procurement shortages. Additionally, poor inventory management in some hospitals can lead to issues such as unsold drugs, inventory buildup, expiry, and tying up hospital funds (12, 24–26).

From the literature, it is evident that existing studies generally agree that the NCDP effectively reduces the purchase prices of listed drugs, lowers the prices of some drugs, increases drug usage, adjusts the income structure of healthcare institutions, and reduces the pricing levels of pharmaceutical companies. However, certain negative effects exist, particularly regarding the coordination of interests among hospitals, governments, and drug manufacturers, which could potentially impact patient interests. Current research evidence on the NCDP is limited to local hospital data, lacking comprehensive studies on national-level medical expenditure. Additionally, quantitative research on whether the NCDP can genuinely limit medical costs is still lacking.

Based on the above analysis, this paper proposes the hypothesis that.

***Hypothesis 1***: NCDP can significantly lower PCHHCE by directly intervening in drug prices.

***Hypothesis 2***: Various household factors will impact NCDP, leading to diverse effects on reducing healthcare costs in urban and rural areas as well as across regions, health insurance types, income brackets, and employment statuses.

## 3 Methods

### 3.1 Data sources

The study utilized the China Family Panel Studies (CFPS) dataset, encompassing 25 provinces, autonomous regions, and municipalities directly governed by the Central Government. The dataset is collected by the Institute of Social Science Survey (ISSS) and captures data at individual, household, and community levels, portraying social, economic, demographic, and educational shifts in China. The database serves as a resource for researchers and policy analysts. The CFPS has been carried out in six periods from 2010 to 2020. Considering the initiation of the NCDP pilot in early 2019, this study employs panel data from 2016, 2018, and 2020 to investigate the impact of the NCDP on *per capita* households. The primary constructed independent variable is whether the family’s prefecture-level city is among the pilot cities involved in the NCDP. The CFPS data encompassed participation from 162 districts and counties at the prefectural and municipal levels. Following data processing, a total of 454 households were included in the district sample, amounting to 1,362 observations.

### 3.2 Model setting and variable definition

A direct approach to assess the NCDP’s impact on PCHHCE involves comparing the difference in *per capita* household health expenditure in the pilot areas pre and post-policy implementation. However, factors beyond the NCDP might influence this variance. Apart from the NCDP impact, various general factors evolving over time could influence this disparity. To mitigate the influence of other factors, this paper adopts a difference-in-difference (DID) approach to analyze the effect of NCDP on PCHHCE. The fundamental principle of this method involves identifying households in prefectures not part of the pooling pilot and utilizing their PCHHCE to mirror the influence of factors apart from the NCDP. The settings can be referred to in Table 1.

**Table 1:** Setting of treatment and control groups in DID.

NCDP	Treat	Control	Diff-1
Before	PCHHCE samples of treatment group households in NCDP pilot areas surveyed by CFPS in 2016 and 2018	PCHHCE samples of control group households outside NCDP pilot areas surveyed by CFPS in 2016 and 2018	Difference in PCHHCE samples between treatment and control group households in 2016 and 2018
After	PCHHCE samples of treatment group households in NCDP pilot areas surveyed by CFPS in 2020	PCHHCE samples of control group households outside NCDP pilot areas surveyed by CFPS in 2020	Difference in PCHHCE samples between treatment and control group households in 2020
Diff-2	Change in PCHHCE for treatment group households in NCDP pilot areas before and after the pilot (including effects of NCDP and other factors)	Change in PCHHCE for control group households outside NCDP pilot areas before and after the pilot (including only the effects of other factors)	Actual NCDP policy effect on the change in household PCHHCE

The reference for model follows the baseline model setup of Yue and Ye (27). The model settings are shown in Equation 1:


(1) $$Y_{ijt}=\beta_{0}+\beta_{1}\mathrm{Trea}t_{jt}+\beta_{2}X_{ij}+\eta_{j}+\gamma_{t}+\varepsilon_{ijt}$$

*i, j* and *t* represent the household that was interviewed, the district within the prefecture where the household is situated, and the time period of the survey (2016, 2018, and 2020) respectively. In this paper, we use $\mathrm{Trea}t_{jt}$ The core explanatory variable is NCDP, indicates whether the prefecture-level city and county *j* where the household is located has been used as a NCDP at the time of the survey in period *t*. In the 2016–2020 China Family Panel Studies, 162 districts and counties under 25 provinces and municipalities directly under the central government were surveyed, of which 31 districts and counties under 11 municipalities were included in this pilot NCDP.

In the realm of data selection, our study employs the data processing methodologies proposed by Qi et al. (28), Xiong et al. (29), and Wang et al. (12). To mitigate variations in medical insurance reimbursement rates across provinces, the research sample will be drawn from households situated in the provinces where the pilot cities engaged in collective procurement are located. Specifically, these samples encompass households surveyed in the China Family Panel Studies (CFPS) from Beijing, Shanghai, Chongqing, Tianjin, the four direct-controlled municipalities, as well as the provinces housing Shenyang, Dalian, Guangzhou, Shenzhen, Chengdu, and Xi’an.

Historically, prior literature commonly employed *per capita* health care expenditure from the “China Statistical Yearbook” or *per capita* health expenditure from the “China Health Statistics Yearbook” as indicators of medical expenses (30, 31). However, *per capita* health care expenditure solely captures the average personal medical expenses and fails to account for individual and group disparities. In alignment with the methodology advocated by Mao and Zhao (32), this paper selects for micro-level medical expenditure data sourced from the China Family Panel Studies (CFPS) database and employs PCHHCE as the metric for individual medical expenses. This methodology is deemed more adept at capturing an individual’s medical expenses within a family context compared to the utilization of national or provincial *per capita* health care expenditure, as observed in previous literature.

As the core explanatory variable of NCDP is the start in 2019, this paper sets the treatment group after the start of the pilot policy $\mathrm{Trea}t_{jt}=1\text{,}$ pilot districts and counties in 2020 $\mathrm{Trea}t_{jt}=1$ ; non-pilot districts and counties in 2020 $\mathrm{Trea}t_{jt}=0$ ; all districts and counties in 2016 and 2018 $\mathrm{Trea}t_{jt}=0$ . Meanwhile, as the decision of whether to include the pilot collection areas is a government decision, and is not influenced by the economic and social status of local residents’ households and consumption habits, the regression can avoid the problem of sample selection bias to the greatest extent. $Y_{ijt}$ denotes the core explained variable, includes the actual value of PCHHCE of respondent household *i* at time *t.* Control variables $X_{ij}$ include the age of the household head, the self-rated health of the household head, the education level of the household head, the average number of years of schooling of the household, the size of the household, the proportion of the household participating in health insurance *per capita*, the household consumption expenditure on education, household chronic diseases, household dependency ratio and the household income *per capita* at the beginning of the period, of respondent household *i*. For the core explained variable and control variables, all of the above variables are divided by the number of household members and then added by one and logged. District and county fixed effects that do not vary over time are controlled for $\eta_{j}$ . The dummy variable $\gamma_{t}$ indicating the number of survey periods *t* controls for time-fixed effects across survey periods. $\varepsilon_{ijt}$ is the error term. Coefficients $\beta_{1}$ denotes the effect of NCDP on the PCHHCE of the district to which it belongs, and is the core parameter of interest.

Moreover, this paper explores the heterogeneity of NCDP’s impact on PCHHCE. Building on the outcomes of the heterogeneity analysis, the study examines whether the effect of NCDP varies based on health care choice, urban–rural distinctions, location, health insurance types, and household income. Hence, the variables “health care choice”, “urban–rural differences,” “health insurance types,” and “household income” are designed as group variables. The “health care choice” variable indicates whether a household member was hospitalized in the preceding year, denoted by 1 for hospitalization and 0 for outpatient services only. The “health insurance types” variable is assigned a value of 1 for households covered by urban employees’ medical insurance and 0 for those under urban and rural residents’ medical insurance.

Additionally, this study creates the “household income” variable, categorized as “low income,” “middle income,” and “high income,” aiming to examine how NCDP affects various household income brackets differently. The regression coefficients’ standard error is clustered at the district and county levels. In terms of fixed effects selection, the decision not to include household fixed effects in the primary regression follows Huang’s (34) approach. With a relatively short panel data (only three periods), controlling for household fixed effects could reduce degrees of freedom and potentially bias the estimation results. (33) posit that the omitted variable “NCDP,” which gauges whether the surveyed city had implemented an NCDP at the survey time, regardless of individual household economic status or consumption habits, primarily operates at the city level and can be captured by city fixed effects in the Differences-in-Differences model. Hence, no additional fixed effects are applied to the respondent households’ circumstances. Apply winsorization to the 1 and 99% percentiles of the continuous variable to mitigate the influence of extreme values. To prevent regression impact caused by substantial household changes, this study omits households from the sample that relocated or changed their city to more accurately capture NCDP’s effects on pilot area households. Table 2 outlines the definitions of key variables and presents descriptive statistical results.

**Table 2:** Descriptive statistics for key variables.

	Variable name	Variable definitions	Observations	Average value	Standard deviation
Dependent variable	*Per capita* household health expenditure	*Per capita* household health expenditure in the past year plus 1, then the natural logarithm of the district	1,362	6.892	1.316
Independent variables	$\mathrm{Trea}t_{jt}$	Whether the prefecture-level city district has been piloted as a NCDP in period t	1,362	0. 364	0. 481
Control variables	Household head characteristics variables				
	Age	Age of head of household	1,362	31.429	12.662
	Self-assessment of health	Level of health derived from the individual household head’s health self-assessment questionnaire	1,362	0.955	0.207
	Years of education	Years of schooling derived from the level of education of the individual head of household	1,362	5.942	5.442
	Household characteristics variables				
	Years of education	Years of schooling per household derived from household members’ educational attainment	1,362	5.536	3.029
	Family size	Total number of people in the household	1,362	3.161	1.414
	Number of health insurance per household	Calculation of *per capita* participation based on the involvement of household members in health insurance	1,362	0.737	0.440
	Household consumption expenditure on education *per capita*	Household education expenditure *per capita* in the past year plus 1, then take the natural logarithm	1,362	6.463	3.079
	Household *per capita* Income	Household income *per capita* for the past year plus 1, then take the natural logarithm	1,362	9.632	1.661
	Household chronic Diseases	Whether there are patients with chronic diseases in the household	1,362	0.265	0.441
	Household dependency Ratio	Ratio of non-working age population to working-age population in the household	1,362	0.314	0.505

## 4 Results

### 4.1 Main regression results

Table 3 presents the impact of the 2019 NCDP on PCHHCE. Columns (1) and (2), without control variables and without controlling for time and location, yield insignificant regression coefficients. However, after accounting for household and head-of-household variables and time and location-fixed effects, significant regression coefficients emerge. They demonstrate that in the NCDP-piloted areas, the policy implementation had a significant impact on local households’ *per capita* health expenditure, showing a coefficient of −0.106 (significant at the 5% level). The literature on the impact of NCDP on PCHHCE, combined with the quantitative research results of this study, collectively confirms the research conclusions (3, 8–11, 14, 16, 17). It is evident that the policy objectives of the NCDP in pilot areas can be achieved, and under the current medical insurance system, the NCDP can reduce PCHHCE to some extent. This study, differing from previous research primarily focused on individual drugs and partial hospital data, validates the actual effectiveness of the NCDP from a broader data perspective.

**Table 3:** Amount of impact on PCHHCE due to the implementation of NCDP.

	Explained variable: *per capita* household health expenditure		
	(1)	(2)	(3)
$\mathrm{Trea}t_{jt}$	−0.172 (0.141)	−0.062 (0.101)	−0. 106** (0.048)
Age of head of household			0.170*** (0.003)
Self-assessed health of household head			−0.897*** (0.105)
Years of education of the head of household			0.006 (0.024)
Average years of schooling in households			−0.005 (0.005)
Family size			0.337*** (0.000)
Number of health insurance per household			1.019*** (0.020)
Household consumption expenditure on education *per capita*			0.093*** (0.000)
Household income *per capita*			0.004 (0.741)
Household chronic diseases			1.945*** (0.224)
Household dependency Ratio			−0.264** (0.130)
Constant term	6.062*** (0.000)	7.159*** (0.014)	5.348*** (0.002)
Year fixed effects	Uncontrolled	Control	Control
Urban fixed effects	Uncontrolled	Control	Control
Sample size	1,362	1,362	1,362
R^2^	0.214	0.190	0.181

Values in brackets are standard regression errors. *, ** and *** represent significance at the 10, 5 and 1% levels, respectively.

### 4.2 Heterogeneity analysis

Despite NCDP demonstrating effectiveness, do households respond differently to the policy shock concerning their healthcare preferences, regional residency, health insurance type, and income levels? This query aids in defining the limits within which NCDP operates. NCDP influences the diversity of PCHHCE concerning healthcare preferences, regional disparities in household locations, the type of health insurance held, and household income levels.

#### 4.2.1 Regional heterogeneity

Yuan et al. (35) discovered that the NCDP led to a decrease in the drug affordability gap between urban and rural areas. The impact of NCDP on noncommunicable disease protection was more pronounced among rural residents compared to their urban counterparts. In 2019, rural residents earned an average of 64.08 RMB per day while urban residents earned 170.16 RMB daily. Workers in rural regions experienced a nearly fourfold increase in NCDP benefits, rising from four (17%) to fifteen (63%) with complete price information. Consequently, the average affordability of drugs reduced from 15.7 days’ pay to 5.3 days’ pay. Conversely, the proportion of affordable medicines for the urban population increased by 30% from 13 (54%) to 18 (75%), with average affordability improving from 5.9 days’ wages to 2.0 days’ wages. Using administrative divisions in the CFPS database, urban and rural households are differentiated based on their residential locations within administrative regions. Urban households reside in urban administrative areas, while rural households reside in rural administrative areas. The results in column (2) of Table 4 demonstrate a significant reduction in PCHHCE for rural families due to NCDP. This study’s findings contradict the conclusion drawn by Tang et al. (7) regarding the significant reduction in healthcare expenditure for urban older adult populations. The reason for this discrepancy may lie in the higher overall medical insurance reimbursement rates for urban households, which could mitigate the significant impact of NCDP drug price fluctuations. However, individual healthcare service expenditures for urban older adult individuals are higher than those for rural older adult individuals, leading to a more noticeable reduction in healthcare expenditure for urban older adult individuals due to the NCDP. Another possible reason could be that households in urban areas are more likely to choose hospitalization when facing medical needs compared to rural households. This results in greater differences in PCHHCE for urban households, leading to higher standard errors for urban areas, which in turn causes the regression results to be non-significant.

**Table 4:** Regional heterogeneity.

	Urban–rural differences	
	(1) City	(2) Rural
$\mathrm{Trea}t_{jt}$	−0.388 (0.414)	−0.290* (0.163)
Constant term	7.762*** (0.001)	8.585*** (0.001)
Control variables	Control	Control
Year fixed effects	Control	Control
Urban fixed effects	Control	Control
Sample size	792	570
R^2^	0.160	0.173

Values in brackets are regression standard errors. *, ** and *** represent significance at the 10, 5 and 1% levels, respectively.

#### 4.2.2 Heterogeneity of health insurance types

In response to the healthcare requirements of urban and rural populations without formal employment, China introduced the New Cooperative Medical System (NCMS) in 2003 and the Medical Insurance for Urban Residents (MIUR) in 2007. The implementation of these two medical insurance systems has been crucial in establishing a robust and comprehensive basic healthcare insurance system. Simultaneously, the enduring urban–rural divide has highlighted the adverse effects of the dichotomy between the two systems, leading to issues of fairness and efficiency, encompassing treatment disparities and duplicated coverage. In 2016, the State Council issued the Opinions on Integrating the Basic Medical Insurance System for Urban and Rural Residents, proposing the amalgamation of the NCMS and MIUR to form a unified healthcare insurance system for Urban and Rural Residents (MIURR). Thus, when categorizing distinct medical insurances, this study aggregates households covered by both NCMS and MIUR into a segment representing MIURR households. The 2021 Statistical Bulletin on the Development of National Medical Insurance Business, published by the National Bureau of Medical Insurance, indicates that within the policy area, the Urban Employees’ Medical Insurance (UEMI) fund covered 84.4% of inpatient expenses. Fund payment ratios for inpatient expenses within Class III, Class II, and Class I, as well as lower-tier medical institutions, were 83.4, 86.9, and 87.9%, respectively. Fund payment ratios for inpatient expenses within Class III, Class II, and Class I, as well as lower-tier medical institutions, were 83.4, 86.9, and 87.9%, respectively. Certain studies indicate that the overall impact of income redistribution through China’s basic medical insurance system is adverse. Medical insurance reimbursement partially mitigates income disparities caused by heightened medical expenses, with the most pronounced effect observed in UEMI, followed by MIUR, and least in the NCMS (25). Clearly, within the actual medical insurance reimbursement process, there exists a discrepancy in the reimbursement ratio between UEMI and MIURR. Thus, the analysis aims to evaluate the impact of NCDP on households with diverse health insurance types.

Based on health insurance management policies, when grouping different types of health insurance, this study combines households covered by the Medical Insurance for Urban Residents (MIUR), New Cooperative Medical System (NCMS), and the healthcare insurance system for Urban and Rural Residents (MIURR) into a single category of healthcare insurance system for Urban and Rural Residents (MIURR) households. Additionally, we distinguish the Urban Employees’ Medical Insurance (UEMI) from the aforementioned group, as there is a significant difference in the reimbursement ratios between these two groups. The findings from Table 5, which categorizes households based on their participation in MIUR and NCMS, demonstrate significant effects of the NCDP in reducing PCHHCE for both MIUR and NCMS households. However, the reduction is more notable among MIUR households, aligning with Tao et al.’s (8) observation regarding the heightened sensitivity of individuals with relatively lower health insurance reimbursement rates to NCDP drug prices. This underscores the NCDP’s effectiveness in lowering PCHHCE for households with higher out-of-pocket ratios, thereby contributing to mitigating existing inequities in China’s medical insurance system’s income redistribution process.

**Table 5:** Heterogeneity of health insurance types.

	(1) Urban Employee Health Insurance	(2) Medical Insurance for Urban and Rural Residents
$\mathrm{Trea}t_{jt}$	−0.065** (0.029)	−0.169* (0.092)
Constant term	3.700*** (0.012)	5.776*** (0.034)
Control variables	Control	Control
Year fixed effects	Control	Control
Urban fixed effects	Control	Control
Sample size	404	958
R2	0.125	0.095

Values in brackets are regression standard errors. *, ** and *** represent significance at the 10, 5 and 1% levels, respectively.

#### 4.2.3 Heterogeneity of household income

The policy objectives of NCDP mirror those of China’s fundamental health insurance system, both striving to ensure “equal opportunities” in benefit provisions through an “equalization” system design. Zhou et al. (36) observed within the health insurance system’s “equalization” framework that benefits favored high-income participants over low-income individuals, uncovering an “inequity under equalization” in the basic health insurance system. In their study, Zhou et al. (37) argue that within rural health insurance, the benefits of medical reimbursement or expenditure reduction tend to primarily favor high-income sick residents, while the impact on the income of low-income residents remains insignificant, leading to an “inverted” scenario where low-income individuals subsidize high-income sick residents.

Is there a variation in the impact of NCDP on PCHHCE across different income levels? The complete household sample is categorized into 3 income groups based on their *per capita* household income in 2018. The entire household sample is categorized into three income groups based on *per capita* household income in 2018: low-income households (0–25% of income levels), middle-income households (25–75% of income levels), and high-income households (75–100% of income levels). Table 6 displays the results obtained from conducting heterogeneity regressions. Columns (2) reveal that NCDP primarily influences households within the 25–75% income bracket, whereas its effect is not significant for families at lower income levels compared to those at higher income levels.

**Table 6:** Household income heterogeneity.

	(1) 0–25% income level	(2) 25–75% income level	(3) 75–100% income level
$\mathrm{Trea}t_{jt}$	−0.106 (0.259)	−0.134*** (0.022)	−0.241 (0.234)
Constant term	6.896*** (0.006)	7.446*** (0.001)	11.672*** (0.000)
Control variables	Control	Control	Control
Year fixed effects	Control	Control	Control
Urban fixed effects	Control	Control	Control
Sample size	177	962	223
R^2^	0.146	0.394	0.495

Values in brackets are regression standard errors. *, ** and *** represent significance at the 10, 5 and 1% levels, respectively.

The phenomenon described may stem from the discrepancy between equal opportunity and “fair outcomes.” In an “equalization” framework, individuals with higher healthcare service utilization rates receive greater reimbursement from medical insurance, leading to increased benefits. However, low-income families with health issues often opt to forego treatment, resulting in significantly lower compensation even if they receive medical services, compared to higher-income groups (37). Consequently, the NCDP policy does not encompass the lowest-income group that abstains from accessing medical services, thereby failing to substantially reduce PCHHCE levels among low-income households.

### 4.3 Robustness tests

#### 4.3.1 Parallel trend test

The prerequisite for policy evaluation using the Difference-in-Differences (DID) method is that the treatment group and the control group must satisfy the parallel trend assumption. In accordance with common practices in the literature, this paper conducts the following event study:


(2) $$Y_{ijt}=\overset{1}{\underset{k=-2,\;k\neq 0}{\sum }}\beta_{k}\mathrm{Treat}\left(k\right)+\beta_{2}X_{ij}+\eta_{j}+\gamma_{t}+\varepsilon_{ijt}$$

**Figure 1:**
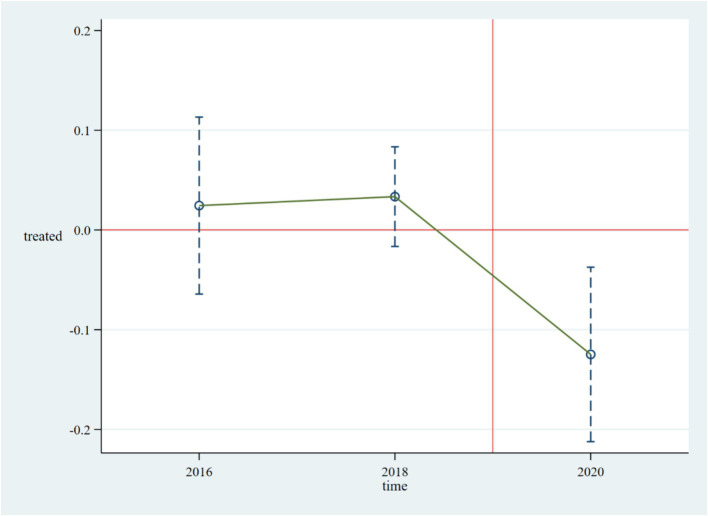
Parallel trend test.

In Equation 2, the variable Treat still indicates whether the district or county in the prefecture-level city has already been designated as an NCDP pilot. $\beta_{k}$ represents the estimated coefficients of the control and treatment groups in the two periods before and one period after the policy implementation. If the coefficients $\beta_{k}$ for the two periods before the policy are not significant, it indicates that the estimated results satisfy the parallel trend assumption. The results of the event study are shown in Figure 1, where the horizontal axis represents the years and the vertical axis represents the estimated coefficients. The hollow circles indicate the policy effects of the NCDP on the treatment group for period k concerning PCHHCE. The point estimates and interval estimates of the average treatment effects for each period are shown in Figure 1. Figure 1 depicts the estimated results of $\beta_{k}$ under the 95% confidence interval. This paper finds that the average treatment effect intervals for $\beta_{k}$ in the two periods before the policy implementation from 2016 to 2018 cross the zero scale line, indicating no significant differences between the treatment and control groups before the implementation of the NCDP pilot policy, thus satisfying the parallel trend assumption. Furthermore, the average treatment effects after the pilot period are far from the zero scale line, and the estimated coefficients $\beta_{k}$ become significant and less than 0 starting from 2020. This indicates that the NCDP had a negative impact on PCHHCE in the pilot areas in 2020, effectively reducing the PCHHCE in these regions.

#### 4.3.2 Permutation test

**Figure 2:**
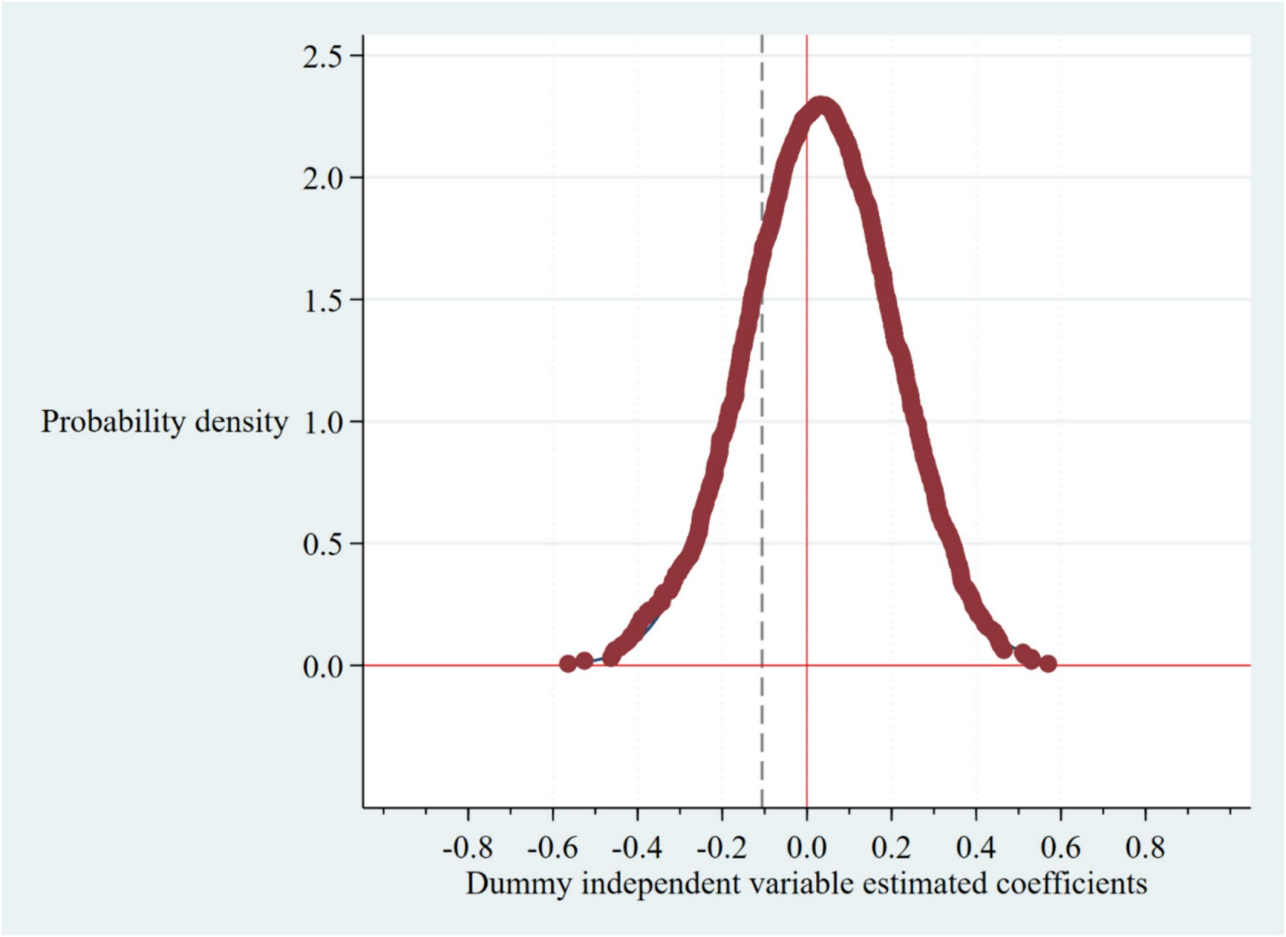
Permutation test.

A permutation test was conducted to eliminate the potential influence of random chance on the primary regression findings. The key explanatory variable, denoted as the dummy variable, representing the policy occurrence location, was formed by random allocation of the introduction time for the set-purchase policy pilot across each prefecture-level municipality. This variable was then integrated into Model (1) to perform regression analysis on PCHHCE, yielding the associated estimated coefficients. Following 1,500 iterations of this process, a graph depicting the distribution of estimated coefficients derived from the regressions was generated. In Figure 2, the vertical line represents the regression coefficient of NCDP on PCHHCE calculated from column (3) of Table 3. Given the negative regression coefficient, indicating statistical significance at the 5% level. The outcomes of the benchmark regression are not influenced by random chance, thereby affirming the reliability of the findings in this study.

#### 4.3.3 Other robustness tests

**Figure 3:**
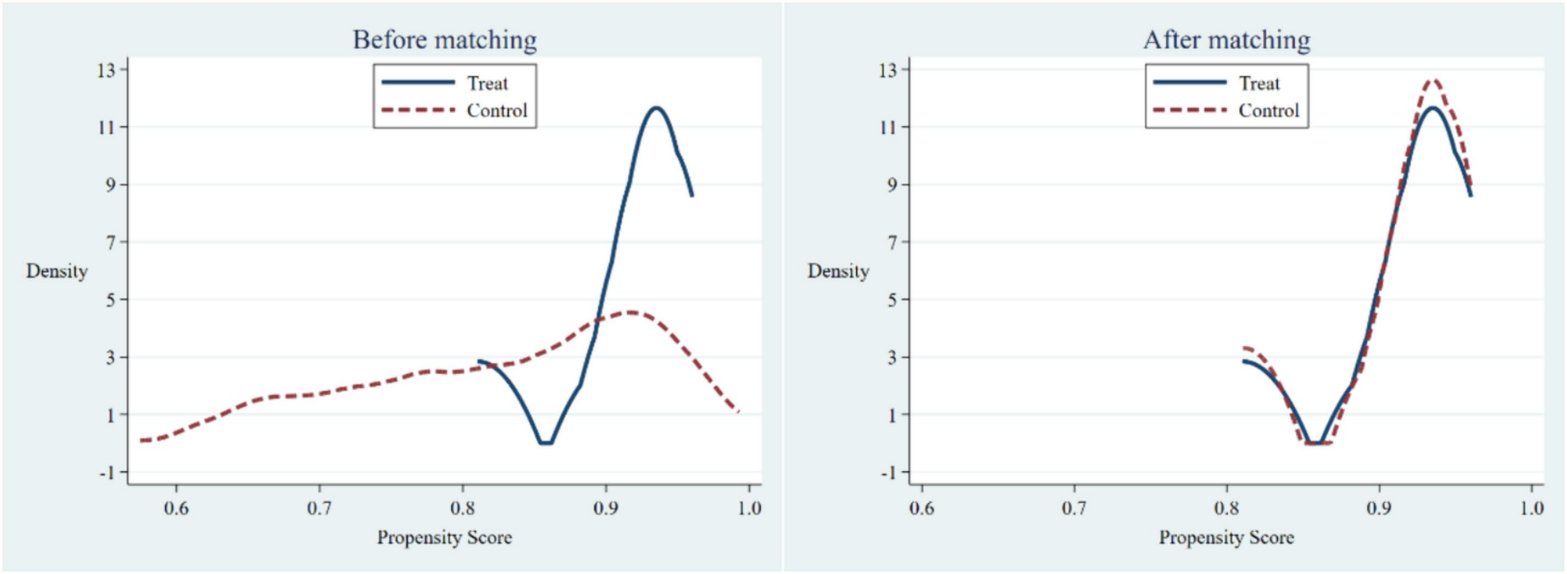
Comparison of propensity scores before and after PSM matching.

The Differences-in-Differences method, used to assess the impact of policy shocks, assumes random selection of the experimental and control groups. Conversely, the categorization of cities in NCDP suggests potential self-selection in the sample. NCDP includes predominantly developed cities in the eastern region and provincial capitals, differing notably from typical prefecture-level towns regarding medical insurance reimbursement rates, drug price concessions, and medical service provision. Consequently, to mitigate the impact of these disparities on estimation results and enhance their robustness, this study utilizes the PSM-DID method. This technique matches propensity scores based on household characteristics for more reliable testing. Employing a 1:2 nearest neighbor matching technique, the matching process resulted in a final sample size of 288 households. Both pre- and post-policy data from the treatment and control groups were preserved, yielding 864 observations. Table 7 displays the discrepancy in means between households involved and not involved in the 4 + 7 policy after the matching process. Notably, no significant differences in any variables are observed after matching, thereby ruling out self-selection of control variables. In Figure 3, the left-hand panel illustrates the variance in propensity scores between the groups before matching, whereas the right-hand panel depicts the difference in propensity scores after matching. The expansion in the common support area between the Treatment and Control groups’ propensity scores indicates compliance with the common support assumption in the PSM matched sample. The findings presented in Table 7 and Figure 3 conclusively affirm the effectiveness of PSM matching in this study and validate that the matched pilot and control groups fulfill the conditions of the parallelism hypothesis.

**Table 7:** Means tests for variables before and after PSM.

Variable name	Unmatched		Matched	
	*t*-value	*p*-value	*t*-value	*p*-value
Age of head of household	−1.09	0.276	−0.47	0.637
Self-assessed health of household head	4.95	0.193	0.37	0.714
Years of education of the head of household	11.23	0.241	0.02	0.981
Average years of schooling in households	10.35	0.605	−0.12	0.904
Family size	−1.78	0.075	0.70	0.481
Number of health insurance per household	2.68	0.007	0.42	0.678
Household consumption expenditure on education *per capita*	13.32	0.060	1.07	0.284
Household chronic diseases	12.85	0.031	1.54	0.306
Household dependency Ratio	−7.35	0.145	0.88	0.381
Household income *per capita*	21.28	0.229	−0.30	0.768

In Table 8, Column (1) presents the empirical outcomes of cross-verified scores following matching. These results affirm a significantly negative impact of NCDP on average household healthcare expenditure, echoing the findings in column (3) of Table 3 and further strengthening the outcomes. Additionally, (38) proposes that re-weighting through semiparametric difference-in-differences can enhance the plausibility of the parallel trends hypothesis in DID, addressing potential differences in characteristics between the experimental and control groups. Consequently, this study employs semiparametric double differences as a robustness assessment. The regression analysis confines the propensity score to households with values ranging from 0.05 to 0.95. The regression outcomes in Column (2) of Table 8 reveal a significant negative effect of NCDP on average household health expenditure at the 0.05 significance level. Furthermore, unobservable factors at the respondent household level could impact average household healthcare expenditure. This study incorporates individual fixed effects at the household level, following Huang et al.’s (33) approach, in Model (1) to mitigate unobservable variables that remain constant within the household over time. The regression findings in Column (3) generally align with the primary regression outcomes, indicating a significantly negative impact of NCDP on PCHHCE when accounting for individual effects.

**Table 8:** Robustness tests.

	(1) PSM-DID	(2) ABS-DID	(3) Individual fixed DID
$\mathrm{Trea}t_{jt}$	−0.113* (0.059)	−0.261** (0.126)	−0.121* (0.062)
Constant term	7.382*** (0.886)	Not given	5.316*** (1.074)
Control variables	Control	Control	Control
Year fixed effects	Control	Control	Control
Urban fixed effects	Control	Control	Control
Sample size	864	969	1,017
R^2^	0.145	Not given	0.138

## 5 Conclusion

This study utilized data from the China Family Panel Studies (CFPS) to examine the impact of the NCDP on PCHHCE. By leveraging the time difference in implementing the NCDP in “4 + 7” pilot cities, the Difference-in-Differences (DID) method was employed for estimation. The research findings, within the framework of a general equilibrium model of healthcare expenditure and health investment, confirmed that the NCDP, implemented through a pre-compensation mechanism, can effectively address certain issues in the current medical insurance system. It promotes fairness in healthcare services across different households and reduces PCHHCE. The “4 + 7” NCDP significantly decreased PCHHCE in the pilot areas. To further analyze the impact of the NCDP on PCHHCE, heterogeneous variables were included in the regression. The study found that the NCDP notably reduced healthcare expenditure in households residing in rural areas versus eastern China, participating in rural and urban medical insurance schemes, and with income levels between the 25th and 75th percentiles.

However, this study also identified several issues with the NCDP. Firstly, the current “4 + 7” NCDP pilot covers a limited range of drug categories, which does not fully address residents’ daily medication needs. Secondly, the pilot areas of NCDP are concentrated in eastern provincial capitals and developed cities, with insufficient promotion in underdeveloped areas in central and western China. Thirdly, NCDP has not completely addressed healthcare inequality issues. In the empirical part of this research, it was found that the lowest-income groups did not effectively reduce their healthcare expenditure, indicating existing problems in institutional design and policy dissemination. Finally, in practical operation, there are coordination deviations between healthcare institutions and government departments, as well as conflicts of interest between healthcare enterprises, affecting patients’ actual medication usage.

The emergence of the aforementioned issues highlights several policy insights for further development of the NCDP. Firstly, expanding the coverage of drug categories and usage scenarios under the NCDP is crucial to ensure the inclusion of drugs that are widely needed by patients. Secondly, prioritizing the implementation of the NCDP in the central and western regions is essential to enhance fairness in healthcare services. Thirdly, strengthening the promotion and publicity of the NCDP is necessary to ensure that all eligible individuals fully benefit from its advantages and to alleviate concerns among low-income families regarding participation in medical services. Lastly, addressing identified policy issues promptly and coordinating the interests of various stakeholders in the medical supply chain are key to ensuring the smooth operation of the policy.

This study can be enhanced and broadened in several ways. Firstly, it should go beyond assessing the impact of the “4 + 7” drug NCDP pilot on PCHHCE. This can be achieved by considering the gradual expansion of coverage areas, drug categories, combination therapy choices, and the inclusion of medical equipment under NCDP, factors that could alter its effect on healthcare expenditure. Secondly, due to data limitations, this study focuses solely on the short-term impact of NCDP on PCHHCE. As the policy implementation period extends, the dynamic effects of NCDP on PCHHCE may evolve, warranting further investigation. Thirdly, delving into the potential influence of NCDP on other categories of household consumption, including nonlinear relationships and heterogeneity, would enrich the analysis. Lastly, exploring the impact of physician-coordinated medication and the long-term effects of NCDP-listed drugs on patient health outcomes in the actual operation of NCDP necessitates longer-term data support, a consideration for future research endeavors.

## Data Availability

Publicly available datasets were analyzed in this study. This data can be found at: http://www.isss.pku.edu.cn/cfps.
